# Effectiveness of Diosmin, Cumarin and Arbutin in Reducing Edema and Pain After Hip or Knee Arthroplasty

**DOI:** 10.3390/jcm15155872

**Published:** 2026-07-27

**Authors:** Andrea Saini, Marco Gramaglia, Simonetta Piano, Alberto Mattioda

**Affiliations:** Physical Rehabilitation Medicine Unit, “Beata Vergine Consolata”—Fatebenefratelli Hospital, 10077 San Maurizio Canavese, Turin, Italy

**Keywords:** osteoarthritis, hip and knee arthroplasty, edema, pain, symptomatology assessment, rehabilitation treatment, deep venous thrombosis

## Abstract

**Background/Objectives**: To assess the efficacy of a product containing Diosmine, Cumarin and Arbutin (Linfadren^®^) in reducing edema and pain and improving specific symptomatology in patients undergoing rehabilitation treatment after hip or knee arthroplasty. **Methods**: In this monocentric prospective study, fifty consecutive patients, hospitalized for rehabilitation, were administered Linfadren^®^ twice a day for 20 days. Limb circumference, pain and specific symptomatology were assessed at baseline and at the end of the treatment; the specific symptomatology was evaluated by means of the Western Ontario and McMaster Universities Arthritis Index (WOMAC), self-administered. **Results**: Baseline and final limb circumference showed a statistically significant difference with 60.42 ± 6.77 cm vs. 53.7 ± 6.37 cm; *p* < 0.0001. Baseline and final pain showed a statistically significant difference with NRS 4.0 ± 1.01 vs. 0.4 ± 0.70—*p* < 0.0001. Baseline and final specific symptomatology differed in a statistically significant manner with the respective scores 37.1 ± 14.4 vs. 18.1 ± 11.4; *p* < 0.0001. Correlations between the decrease in limb circumference, pain and specific symptomatology showed statistical significance. **Conclusions**: The product containing Diosmine, Cumarin and Arbutin demonstrates efficacy in reducing edema and pain and in improving specific symptomatology in patients undergoing rehabilitation treatment after hip or knee arthroplasty. This can translate to patients’ better ability to perform intensive physiotherapy protocols, thus enhancing the recovery.

## 1. Introduction

Osteoarthritis (OA) is a leading cause of pain and disability worldwide, with the hip and knee being the most commonly affected major joints. These forms of OA lead to individual suffering, reduced quality of life, and increased healthcare costs. According to a very recent study, the pooled prevalence of hip OA in Europe is estimated at 6%, while knee OA prevalence is higher, at 10% [1]. Joint replacement surgery is a common treatment used to address severe arthritis and is widely recognized as the most effective treatment for this pathology, significantly improving outcomes for arthritis patients [2]. However, even after successful surgery, the immediate postoperative phase is often characterized by pain, inflammatory swelling, and temporary functional limitations; these factors can influence the progress of rehabilitation. Despite substantial technical advances in surgical procedures and developments in prosthetic design in recent years, the postoperative phase remains a critical period in which pain, inflammation, and functional impairment may negatively affect rehabilitation outcomes [3]. During the postoperative period, the inflammatory response to surgical procedures plays an essential role in tissue repair but may also become disproportionate, leading to excessive edema, uncontrolled pain, and functional limitations that delay recovery [4]. Untreated chronic swelling could exacerbate discomfort and lead to difficulties in mobility and flexibility [5]. Moreover, edema potentially increases the risk of infection in the impacted region, reduces the flow of blood, and impacts the elasticity of the arteries [6]. Individuals are greatly affected by these complaints, causing both physical and psychological harm. Usually, nonsteroidal anti-inflammatory drugs (NSAIDs) are administered in order to control post-surgical inflammation and this represents a fundamental component of care, integrated within rehabilitation programs. However, the prolonged use of NSAIDs and other analgesic agents is often restricted by their potential adverse effects, particularly gastrointestinal, renal, and cardiovascular complications, which are of greater concern in older adults and in individuals with multimorbidity [7,8]. This scenario has stimulated growing interest in ‘natural’ anti-inflammatory molecules capable of modulating the inflammatory process with minimal or absent side effects. Some studies have been conducted about the efficacy of these agents in various settings [9,10,11].

A product containing Diosmine, Cumarin and Arbutin (Linfadren^®^, Omega Pharma srl) has been commercially available for several years, with the indication for the treatment of lymphatic and venous edema. Some studies have demonstrated its efficacy in the management of patients with a breast cancer-related lymphedema [12], in reducing post-trauma/surgery persistent hand edema [13], and in reducing persistent posttraumatic/postsurgery ankle edema [14].

Diosmin belongs to a group of glycosylated flavonoids and was first isolated from the plants of the Rutaceae family. It is composed of diosmetin (aglycone) and a sugar part, which is the disaccharide 6-O-L-rhamnosyl-D-glucose. This flavonoid, as a phlebotonic drug, is able to improve the condition of the veins with its anti-inflammatory and antioxidant properties. Diosmin is also able to increase venous tone, reduce the inflammatory response and capillary leakage, and improve microcirculation [15]. A recent placebo-controlled study has proven single-agent Diosmin as effective in reducing lower-extremity swelling and pain during motion in patients who underwent total knee arthroplasty [16].

Cumarin (5–6 benzo-a-pyrone) plays a role in reducing edema by activation of proteolytic activity of macrophage. In lymphedema, many macrophages are present, but their activity is greatly reduced; some studies provide evidence that benzopyrones like coumarin are able to revert this state. In addition, Cumarin reduces inflammation and exerts other actions including the reduction in excess fibrosis along with the excess interstitial protein and the excess numbers of new blood and lymph vessels [17].

Arbutin (4-hydroxyphenyl-β-D-glucopyranoside, β-arbutin), a naturally occurring β-D-glucopyranoside derivative of hydroquinone primarily extracted from the leaves of bearberry plant *Arctostaphylos uva-ursi*, exhibits multifaceted bioactivities, including antioxidant, anti-inflammatory, antimicrobial, antihyperglycemic, and antitumor properties [18]. Clinically, Arbutin is used in drugs for the treatment of lower urinary tract infections and as a food supplement, whereas α-arbutin is primarily used as a dermatological depigmenting agent [19]. Studies in animal models provide evidence of its dose-dependent activity in decreasing post-ischemia vascular injury, capillary damage, and cell death by inhibiting inflammation [20,21].

The purpose of this study is to investigate the efficacy of Linfadren^®^, when used in addition to conventional treatment, in reducing edema, pain and improving patients’ specific symptomatology, consisting of the questions of the WOMAC questionnaire which is reported in detail in the Material and Methods, in patients who underwent total hip or knee replacement for severe OA, together with rehabilitation-specific therapeutic exercise in patients hospitalized for intensive rehabilitation treatment. The primary end point is to explore a reduction in the circumference of the surgical treated limb, and secondary end points are to explore a reduction in pain and an improvement in patients’ symptomatology as evaluated by means of a standardized, validated questionnaire (WOMAC).

## 2. Materials and Methods

### 2.1. Subjects and Setting

Consecutive patients, who underwent total hip arthroplasty or total knee arthroplasty with scheduled intervention, for hip or knee osteoarthritis, hospitalized at Physical and Rehabilitation Unit of “Beata Vergine Consolata”—Fatebenefratelli Hospital for intensive rehabilitation treatment, were included in the study. The eligibility criteria for the inclusion in the study were remarkable edema, age > 18, ability to perform tests, and absence of clear signs of surgical wound infection. The exclusion criteria were illiteracy, relevant psychiatric disorders (i.e., schizophrenia, bipolar disorder, and major depression) drug addiction, and major illnesses (i.e., uncompensated COPD, advanced heath failure, advanced renal failure, and advanced liver failure).

Each patient was advised that they would be administered Linfadren^®^, a product currently commercially available for the treatment of lymphatic and venous edema, in order to reduce the edema and decrease pain and would be requested to compile a questionnaire focused on specific symptomatology. After the explanation, the patients were asked to sign a detailed informed consent. All procedures followed the principles outlined in the Declaration of Helsinki.

Patients were screened for the study at their admission to the Physical and Rehabilitation Unit, at the same time physiotherapy treatment started, usually three days after surgical intervention of arthroplasty.

### 2.2. Interventions

All patients included in the study were administered Linfadren^®^ (Omega Pharma S.p.A., Cantù, Italy) twice a day, far from food intake, for 20 days, beginning the first day of hospitalization at the Physical and Rehabilitation Unit.

Patients were concomitantly administered a standard analgesic therapy consisting of Paracetamol 1000 mg twice a day, in the morning and evening, for 10 days, together with Ibuprofen 600 mg once a day at noon, for 5 days.

All the patients received anti-thromboembolic prophylaxis with low-molecular-weight heparin, as prescribed by current guidelines in major orthopedic surgery.

The usual medication regimen for specific concomitant pathologies (mainly arterial hypertension, hyperlipidemia, mild COPD, and non-insulin-dependent diabetes mellitus), already taken before surgical intervention, was administered under medical supervision.

The patients followed a physiotherapy protocol consisting of progressive passive and active range of motion increase, progressive muscle reactivation, soft tissues mobilization once incision and scar are closed, proprioceptive training to improve body/spatial awareness of the operative extremity, gait training on flat surfaces with appropriate device—than discontinued, and functional training to promote independence with activity of daily life (ADLs) and instrumental activities of daily life (IADLs). Each patient performed 3 h of individual physiotherapy treatment daily.

### 2.3. Outcome Assessments

One of the physicians belonging to the Physical and Rehabilitation Medicine Unit performed the measurement of the circumference of the operated limb at baseline and at the end of the treatment. The anatomic landmarks were, for hip arthroplasty, 5 cm under the gluteal fold and, for knee arthroplasty, 10 cm below the center of the patella. Moreover, each patient was asked at baseline and at the end of the treatment what intensity of pain was felt, as expressed as a numeric rating scale (NRS), ranging from 0 to 10, 0 being the absence of pain and 10 the worst pain the patient could experience.

The Western Ontario and McMaster Universities Arthritis Index (WOMAC) questionnaire was provided at baseline and at the end of the treatment and self-administered. A physician was available for answering questions about compiling the questionnaire. Subgroup analysis of hip arthroplasty and knee arthroplasty was pre-planned.

### 2.4. WOMAC Questionnaire

The Western Ontario and McMaster Universities Arthritis Index is widely used in the evaluation of hip and knee osteoarthritis. It is a self-administered questionnaire consisting of 24 items divided into 3 subscales, as follows: pain (5 items): during walking, using stairs, in bed, sitting or lying, and standing upright; stiffness (2 items): after first waking and later in the day; and physical function (17 items): using stairs, rising from sitting, standing, bending, walking, getting in/out of a car, shopping, putting on/taking off socks, rising from bed, lying in bed, getting in/out of bath, sitting, getting on/off toilet, heavy domestic duties, and light domestic duties. Each item contains 5 possible answers from which the patient chooses for their self-evaluation. The test questions are scored on a scale of 0–4, which correspond to none (0), mild (1), moderate (2), severe (3), and extreme (4). The scores for each subscale are summed up, with a possible score range of 0–20 for pain, 0–8 for stiffness, and 0–68 for physical function. Higher scores on the WOMAC indicate worse pain, stiffness, and functional limitations. Usually, the sum of the scores for all three subscales give a total WOMAC score that is employed [22]. The WOMAC is available in over 65 languages, including Italian, and has been linguistically validated [23].

As the patients involved in the study were hospitalized and did not do some of the activities mentioned in the questionnaire, the items concerning getting in/out of a car or bus, go shopping, do light domestic duties, and do heavy domestic duties, were excluded from the evaluation. The questionnaire has been therefore mentioned in the paper as the “modified WOMAC”.

### 2.5. Statistical Analysis

Data were processed using MedCalc © (version 23, 2025 MedCalc Software Ltd., Ostend, Belgium). Normal distribution of the data was assessed by means of the Kolmogorov–Smirnov test. When a normal distribution emerged, a *t*-test for paired samples was employed. When the distribution was non-normal, Wilcoxon test for paired samples was employed. Correlation tests were also performed. All tests were two-sided. A *p* < 0.05 was considered as statistically significant. Box and Whiskers data comparison graphs were used to show the differences form baseline to final assessments.

## 3. Results

From November 2024 to September 2025, 50 consecutive patients were enrolled in the study, out of 53 screened, of whom 3 declined the invitation.

The median age was 71.5 years (range 54–83). Concerning the gender, the patients included 19 men and 31 women. Of the whole patient population, 15 underwent hip arthroplasty and 35 underwent knee arthroplasty.

**Figure 1:**
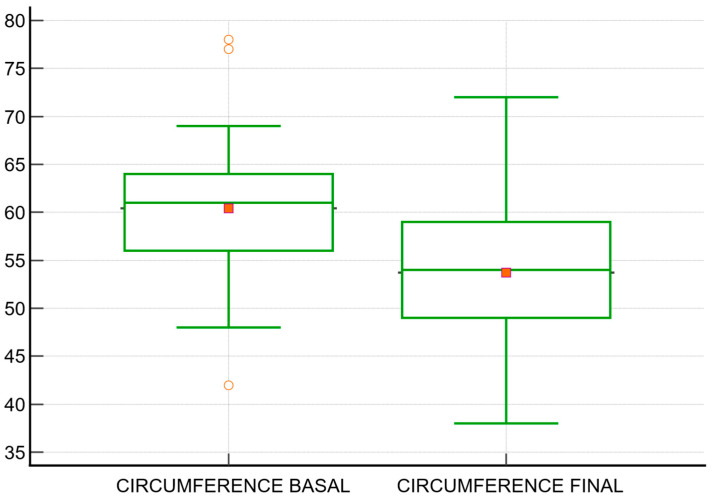
Baseline and final limb circumference in the whole patient population. *p* < 0.0001.

The comparison between baseline limb circumference and final limb circumference shows a statistically significant difference with 60.42 ± 6.77 cm vs. 53.70 ± 6.370 cm; *p* < 0.0001. This result is represented graphically in Figure 1.

**Figure 2:**
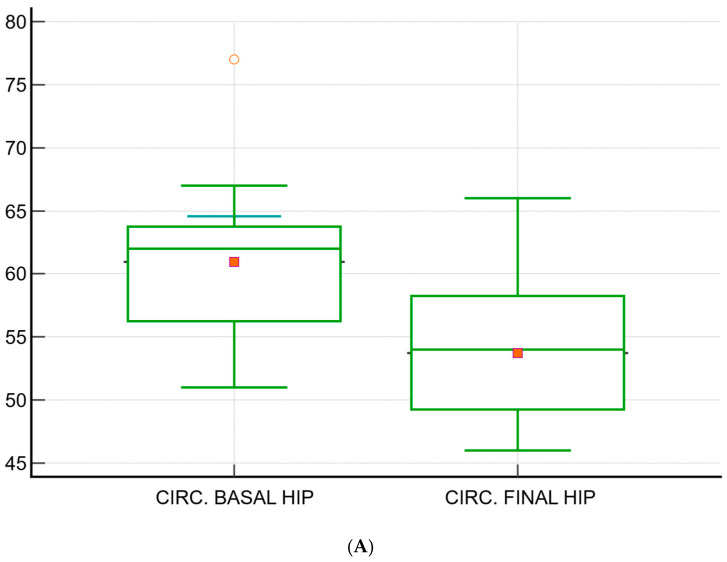

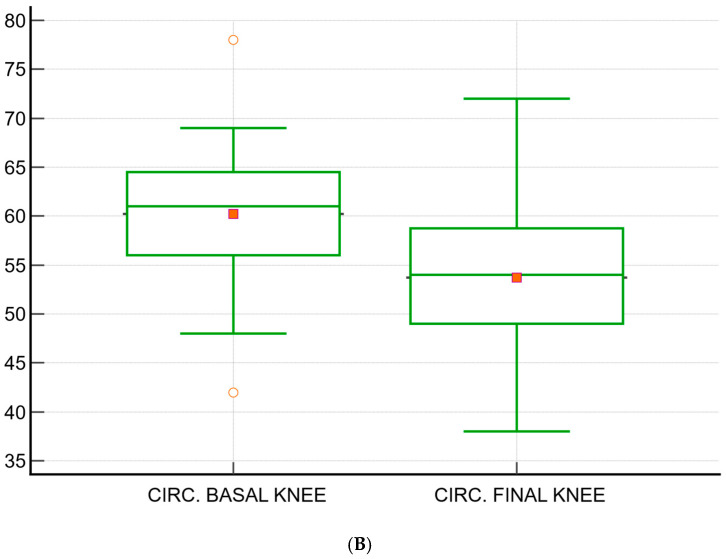
(**A**) Baseline and final limb circumference in the subgroup of patient with hip arthroplasty. (**B**) Baseline and final limb circumference in the subgroup of patient with knee arthroplasty. *p* < 0.0001.

When dividing into the two subgroups, hip arthroplasty patients and knee arthroplasty patients, according to the pre-planned analysis, a statistically significant difference from baseline to final limb circumference is maintained as follows. Hip arthroplasty patients: 60.93 ± 6.58 cm vs. 53.73 ± 5.73 cm—*p* < 0.0001. Knee arthroplasty patients: 60.21 ± 6.93 cm vs. 53.68 ± 6.71 cm—*p* < 0.0001. These results are represented graphically in Figure 2A,B.

**Figure 3:**
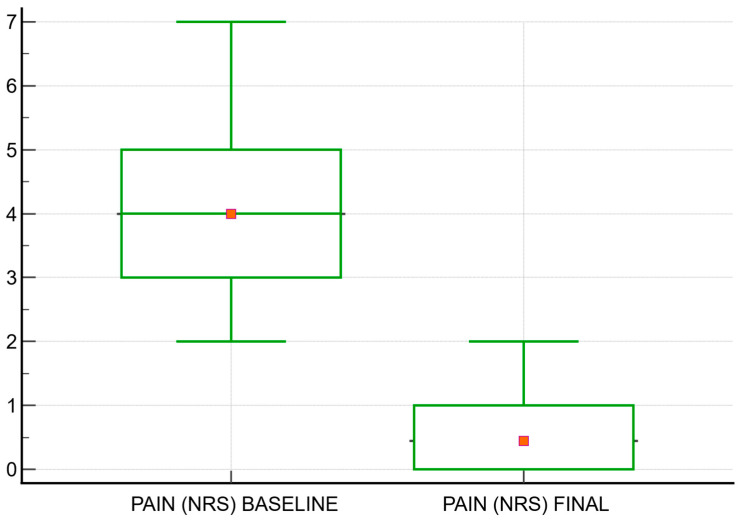
Baseline and final pain in the whole patient population. *p* < 0.0001.

The comparison between baseline pain and pain at the end of the treatment shows a statistically significant difference with NRS 4.0 ± 1.01 vs. 0.4 ± 0.70—*p* < 0.0001. This result is represented graphically in Figure 3.

When dividing into the two subgroups, hip arthroplasty patients and knee arthroplasty patients, according to the pre-planned analysis, a statistically significant difference from baseline to final pain is maintained as follows. Hip arthroplasty patients: NRS 3.8 ± 0.86 vs. 0.06 ± 0.25—*p* < 0.0001. Knee arthroplasty patients: NRS 4.08 ± 1.06 vs. 0.33 ± 0.77—*p* < 0.0001.

**Figure 4:**
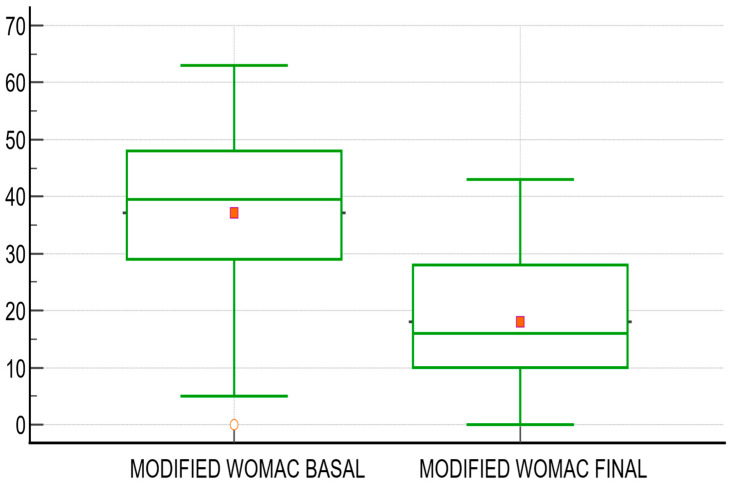
Baseline and final total WOMAC score in the whole patient population. *p* < 0.0001.

Lastly, the comparison between baseline and final modified WOMAC questionnaire score shows a statistically significant difference with 37.1 ± 14.4 vs. 18.1 ± 11.4—*p* < 0.0001. This result is represented graphically in Figure 4.

When dividing into the two subgroups, hip arthroplasty patients and knee arthroplasty patients, according to the pre-planned analysis, a statistically significant difference from baseline to final WOMAC questionnaire scores is maintained as follows. Hip arthroplasty patients: 35.0 ± 16.25 vs. 12.6 ± 9.7—*p* = 0.0001. Knee arthroplasty patients: 38.1 ± 13.7 vs. 20.4 ± 11.4—*p* < 0.0001.

Moreover, correlation tests were performed: a statistically significant correlation was found between a reduction in limb circumference and a reduction in pain—NRS (*p* < 0.0001), between a reduction in limb circumference and modified WOMAC score reduction (*p* < 0.0001), and between a reduction in pain—NRS and modified WOMAC score reduction (*p* = 0.02).

Noteworthy, no deep venous thrombosis event occurred in the study population.

No relevant adverse events, particularly potentially related to Linfadren^®^ administration, was recordered.

## 4. Discussion

The study reached its primary end point as well as its secondary end points, providing evidence that Linfadren^®^ is able to significantly reduce edema and pain and to improve specific symptomatology in patients who underwent hip or knee arthroplasty for osteoarthritis. The end points are reached even when dividing the study population into the two subgroups—hip arthroplasty patients and knee arthroplasty patients—according to the pre-planned subgroup analysis.

Linfadren^®^ has been commercially available for several years with the indication for the treatment of lymphatic and venous edema, but its efficacy had never been documented in patients with edema following hip or knee arthroplasty. In our study the administration of Linfadren^®^ in these patients led to a significant improvement in edema, pain and specific symptomatology. This improvement translates to an increased ability of the patients to perform intense rehabilitation treatment, which could be difficult with more pain and stiffness.

Linfadren^®^ is a product containing natural compounds; in recent decades, a rising interest has been developed in ‘natural’ anti-inflammatory molecules capable of modulating the inflammatory process with minimal or absent side effects for various clinical situations.

A recent study provided data that administration of bromelain, troxerutin, and escin supplementation was associated with reductions in postoperative pain and edema and with faster functional recovery in a population of adults aged 65 years or older, admitted to a post-acute rehabilitation unit after total hip or knee arthroplasty [9]. This study however lacks an evaluation of specific symptomatology by means of a validated questionnaire administered to the patients. Other studies provided evidence about the efficacy of various natural compounds, like flavonoids, O-(β-hydroxyethyl)-rutosides, and red vine leaf extract AS 195, in chronic venous insufficiency [24,25,26]. Furthermore, a product containing Trypsin, bromelain and rutoside obtained a remarkable reduction in pain, inflammation, and inflammatory biomarker levels after orthopedic surgery [27].

These data support the hypothesis that natural molecules are active in the inflammatory processes leading to edema and pain; the results of our study are in line with these findings.

After orthopedic surgery, venous and lymphatic insufficiency, hematoma and joint effusion are the usual phenomena, which play an important role in the development of limb edema. The components of the investigated product, as mentioned in the Introduction, have demonstrated the ability to improve the condition of the veins with anti-inflammatory and antioxidant properties, increase venous tone and reduce the inflammatory response and capillary leakage [15]; these properties could make Linfadren^®^ helpful in reducing these clinical consequences of surgery. Moreover, the activity in reducing inflammation, reducing excess fibrosis and the excess interstitial protein, and decreasing post-ischemia vascular injury, could play a role in improving muscle condition; however, it must be underlined that this is only a hypothesis. Lastly, adipose tissue accumulation is common; some in vitro studies suggest that macrophages are involved in adipose tissue pathophysiology in the context of OA [28]; the mechanisms of this activity are very complex and any consideration in regards to any role of Linfadren^®^ would be scarcely consistent.

In the present study, an analgesic standard treatment was administered to all patients, according to clinical practice. This treatment enhances pain reduction; however, the NSAID (Ibuprofen) was administered once a day, only for 5 days, and Paracetamol twice a day, for 10 days. This represents a substantially mild analgesic treatment, concomitant with Linfadren^®^ administration for 20 days.

Despite standard thromboprophylaxis, symptomatic breakthrough venous thromboembolism, primarily distal deep venous thrombosis, still developed in patients undergoing major orthopedic surgery, with percentages widely ranging among the different studies [29,30]. In the study patient population, no deep or superficial venous thrombosis event occurred. Although the study was not designed with this goal, the reduction in the inflammatory response and capillary leakage, as well as the improvement in microcirculation, that Linfadren^®^ components are able to perform, could have some role in preventing thrombosis events. This is currently only a hypothesis; specific studies are needed to investigate the issue.

Limb circumference was measured by one of the physicians belonging to the Physical and Rehabilitation Medicine according to anatomic landmarks. The measure was taken each time by a specific physician. The single-operator measurement could represent a potential weakness, but the anatomic landmarks were clearly established, thus limiting the interobserver variability.

Strengths of this study are the prospective design, the use of clinical standardized tools together with a widely validated specific assessment tool administered to the patients (WOMAC questionnaire), the real-world scenario of postoperative rehabilitation treatment with codified physiotherapy protocols and the adherence to Linfadren^®^ treatment, warranted by the administration by nurses, as the patients were hospitalized.

The study presents a main limitation for the lack of a control group. Edema, pain and specific symptomatology are expected to improve naturally during the postoperative recovery. However, we think that the significant improvements obtained for all the outcomes, in a 20-day period, represent a remarkable result that encourages further investigations in larger clinical studies with a control group or randomization. Further limitations include the relatively small patient population and the monocentricity of the study.

## 5. Conclusions

Notwithstanding some limitations, mainly the lack of a control group, this study suggests that Linfadren^®^ is effective and safe in reducing edema and pain as well as in improving specific symptomatology in patients undergoing rehabilitation treatment after hip or knee arthroplasty for osteoarthritis. By reducing edema and pain, and improving specific symptoms, patients can be able to better perform the physiotherapy protocols, thus enhancing the recovery. On the basis of the results of this study, further multicentric studies involving large patient populations are recommended.

## Data Availability

The datasets generated during and analyzed during the current study are available from the corresponding author on reasonable request.
